# Hyperbaric Oxygen Therapy for Enhanced Postoperative Recovery in Hair Transplantation

**DOI:** 10.7759/cureus.99635

**Published:** 2025-12-19

**Authors:** Flavio Giardiello, Leonardo De Medeiros Quirino, Renan Brigante, Maxim Chumak

**Affiliations:** 1 Hyperbaric Medicine/Hair Restoration Surgery, School of Medical Sciences of Santa Casa de Sao Paulo, Piracicaba, BRA; 2 Medicine, Universidade Federal de Uberlândia, Uberlandia, BRA; 3 Surgery, Grajau General Hospital, São Paulo, BRA; 4 Hair Restoration, Maxim Medical, Fort Lauderdale, USA

**Keywords:** androgenetic alopecia, crusting, follicular unit excision, follicular unit extraction, hair transplantation, hyperbaric oxygen thetapy, patients satisfaction, postoperative recovery, postoperative recovery enhancement, ­wound healing

## Abstract

Postoperative recovery following hair transplantation procedures often involves prolonged healing periods, which can delay patients' return to normal activities. The objective of this work is to evaluate the efficacy of hyperbaric oxygen therapy (HBOT) as an adjunctive treatment in accelerating wound healing, reducing scab permanence, and enabling earlier resumption of standard hygiene practices and daily activities in patients undergoing hair transplantation procedures. Five male patients (age range 28-56 years) undergoing follicular unit excision (FUE) hair transplantation were enrolled in this case report. HBOT was initiated 4-6 hours postprocedure, with daily 90-minute sessions at 2.4 absolute atmospheres for six consecutive days. Standardized assessments included crusting/scabbing (0-4 scale), time to resolution of crusting, graft integration, pain (visual analog scale), patient satisfaction, recovery time, and complications. Complete elimination of scab formation was achieved within five days (range 3-5 days), representing a significant improvement compared to standard postoperative care protocols. Graft integration rates were excellent (97-99%). Patient satisfaction was high (4-5 on a 5-point Likert scale), and recovery time averaged 2.8 days. Pain scores diminished rapidly, with most patients reporting no pain by day three. No complications were observed in any patient. HBOT appears to be a promising adjunctive treatment in the postoperative management of hair transplantation patients. Although more extensive controlled studies are needed to establish optimal protocols, these preliminary findings provide a compelling rationale for the continued exploration of HBOT as a valuable component in improving outcomes and patient experience following hair transplantation procedures.

## Introduction

Hyperbaric oxygen therapy (HBOT) involves breathing 100% oxygen at increased atmospheric pressures to elevate oxygen levels in plasma and tissue. Used in medicine for centuries, it has recently gained significant clinical attention. Research demonstrates HBOT's benefits include combating hypoxia and infection, stimulating capillary regeneration, and reducing ischemia-reperfusion injury. Studies also indicate that HBOT can prevent necrosis or reduce the size of necrotic tissue, establishing a foundation for further research in this area [1-4].

HBOT was employed as an adjunctive treatment in the immediate postoperative period following hair transplantation procedures. This intervention was associated with expedited wound healing, facilitating earlier initiation of postoperative hygiene protocols and accelerated return to patients' normal daily activities [5].

The administration of HBOT in the postoperative phase has demonstrated significant clinical benefits [6,7]. Most notably, the therapy appeared to enhance the rate of epithelialization and reduce inflammatory responses at the recipient and donor sites. This accelerated healing profile permitted the implementation of more aggressive postoperative cleansing protocols without compromising graft viability or wound healing.

The objective of this work is to evaluate the efficacy of HBOT as an adjunctive treatment in accelerating wound healing, reducing scab permanence, and enabling earlier resumption of standard hygiene practices and daily activities in patients undergoing hair transplantation procedures.

## Case presentation

This case report evaluates the effects of hyperbaric oxygen therapy (HBOT) on postoperative recovery following hair transplantation procedures. The study was conducted at Giardiello's Hair Transplant Clinic between October 2024 and January 2025. This study was approved by the Brazilian National Research Ethics Committee system (CEP/CONEP), under CAAE number 93996725.2.0000.0243. All procedures were conducted in accordance with the ethical standards and with the principles of the Declaration of Helsinki and its subsequent amendments.

Five patients (all males, age range 28-56 years) undergoing follicular unit excision (FUE) hair transplantation were enrolled in the study after providing written informed consent. The inclusion criteria comprised: candidates for FUE transplantation requiring 1,800-5,100 grafts; no previous hair restoration procedures; absence of contraindications to HBOT; willingness to comply with the follow-up protocol. The exclusion criteria were: uncontrolled claustrophobia, active smoking, uncontrolled diabetes mellitus, use of anticoagulants that could not be suspended, and active scalp conditions.

All procedures were performed by the same surgical team using standardized FUE techniques. Local anesthesia consisting of lidocaine 2% with epinephrine 1:100,000 was administered. Follicular units were harvested from the occipital donor area using 0.8-1.0 mm punches. Grafts were stored in a hypothermic holding solution (Ringer's lactate with added adenosine triphosphate) at 4°C until implantation. The implantation method employed involved Lion implanters with diameters of 0.8 mm and 1.0 mm.

HBOT was initiated within 4-6 hours after the procedure, with patients treated in a monoplace hyperbaric chamber following a standardized protocol. The sessions were conducted at a pressure of 2.4 atmospheres absolute (ATA) for 90 minutes without air breaks, administered once daily for six consecutive days. A free delivery system provided 100% oxygen, and compression and decompression were performed at a rate of 0.1 ATA per minute [8]. Vital signs were monitored throughout each session by a trained hyperbaric technician. Any adverse events or discomfort were documented.

All patients received standardized postoperative instructions and medications, which included oral antibiotics (cefadroxil 500 mg twice daily) for five days, analgesics as needed for pain control, and application of thermal water spray to the recipient area every two hours while awake during the first 72 hours.

Patients were instructed to sleep with their head elevated at 45° for the first week. A specialized washing protocol was implemented beginning on day one post-procedure, with medical staff supervising daily washing during the HBOT treatment period.

The hygiene protocol commences on the first postoperative day with the application of ozonated water (10-40 μg/mL) via gentle nebulization, administered twice daily to promote antimicrobial effects and stimulate microcirculation. Following this initial phase, we utilized a sulfate-free and paraben-free neutral shampoo (pH 5.5-6.5), forming a lather that was applied with light digital contact while avoiding abrupt movements.

On the second postoperative day, the process was repeated with the introduction of gentle digital movements, followed by the application of a healing oil after each cleansing session, with a contact duration of 10-15 minutes. During days three, four, and five, we introduced gentle circular movements repeated multiple times, alternating between ozonated water, neutral shampoo, and healing oil applications.

The protocol included monitoring through standardized photographic documentation and quantitative assessment of erythema, edema, and crust formation [9,10]. Concentrations were adjusted according to individual patient response and the specific transplantation technique employed.

The following parameters were assessed, and standardized photographic documentation was performed throughout the study (Table 1).

**Table 1:** Parameters assessed and timeline

Parameter	Measurement Method	Assessment Timeline
Crusting/Scabbing	0-4 scale (0=none, 4=severe) [9,10]	Days 1, 3, 5, and 7
Resolution of Crusting	Time to complete resolution	Measured in days
Graft Integration	Visual count of visible follicular units	Day 7
Pain Assessment	10-point analog scale [11]	Daily for the first week
Patient Satisfaction	5-point Likert scale [12]	Day 14
Recovery Time	Time to return to normal daily activities and work	Measured in days
Complications	Documentation of any adverse events	Throughout the study period

Standardized photography was performed at baseline, immediately post-procedure, and on days one, three, and five. All photographs were taken with the same camera (digital SLR with macro lens capability), under standardized lighting conditions, and at consistent distances and angles to ensure comparability across time points and between patients. All assessments were conducted by the same two evaluators, who were experienced in hair transplantation outcomes.

**Figure 1:**
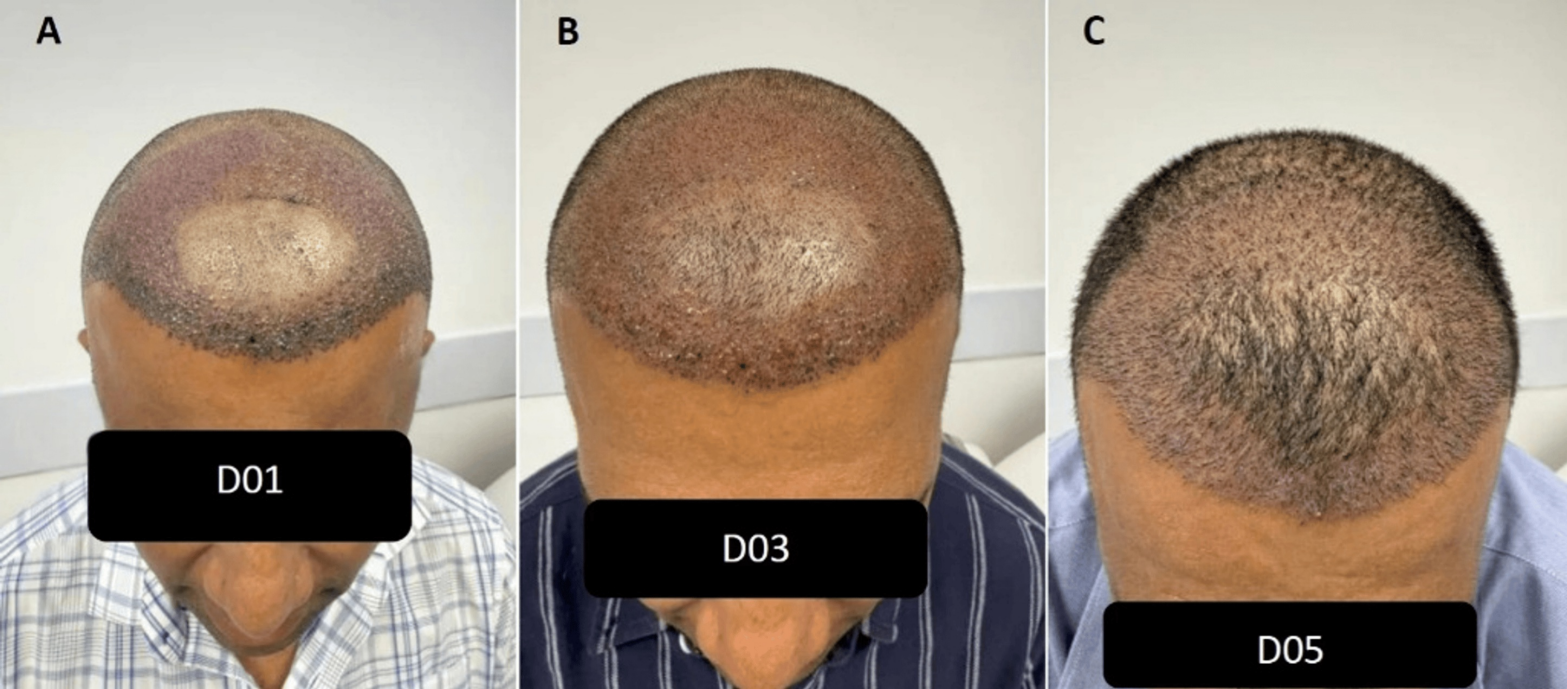
Case A (A) Day 1 postoperatively, showing moderate crust formation and expected erythema. (B) Day 3, marked reduction in crusting and edema. (C) Day 5, near-complete resolution of crusts and satisfactory graft integration.

**Figure 2:**
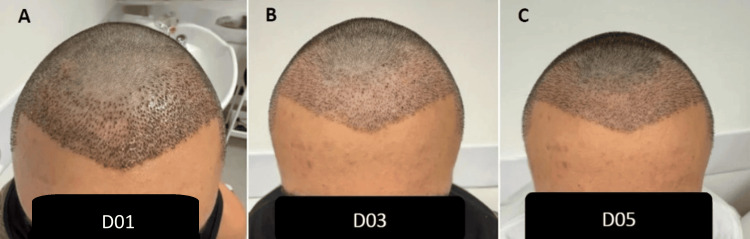
Case B (A) Day 1, mild crusting at recipient sites. (B) Day 3, complete resolution of visible crusts. (C) Day 5, clean recipient area with excellent graft stability and minimal residual erythema.

**Figure 3:**
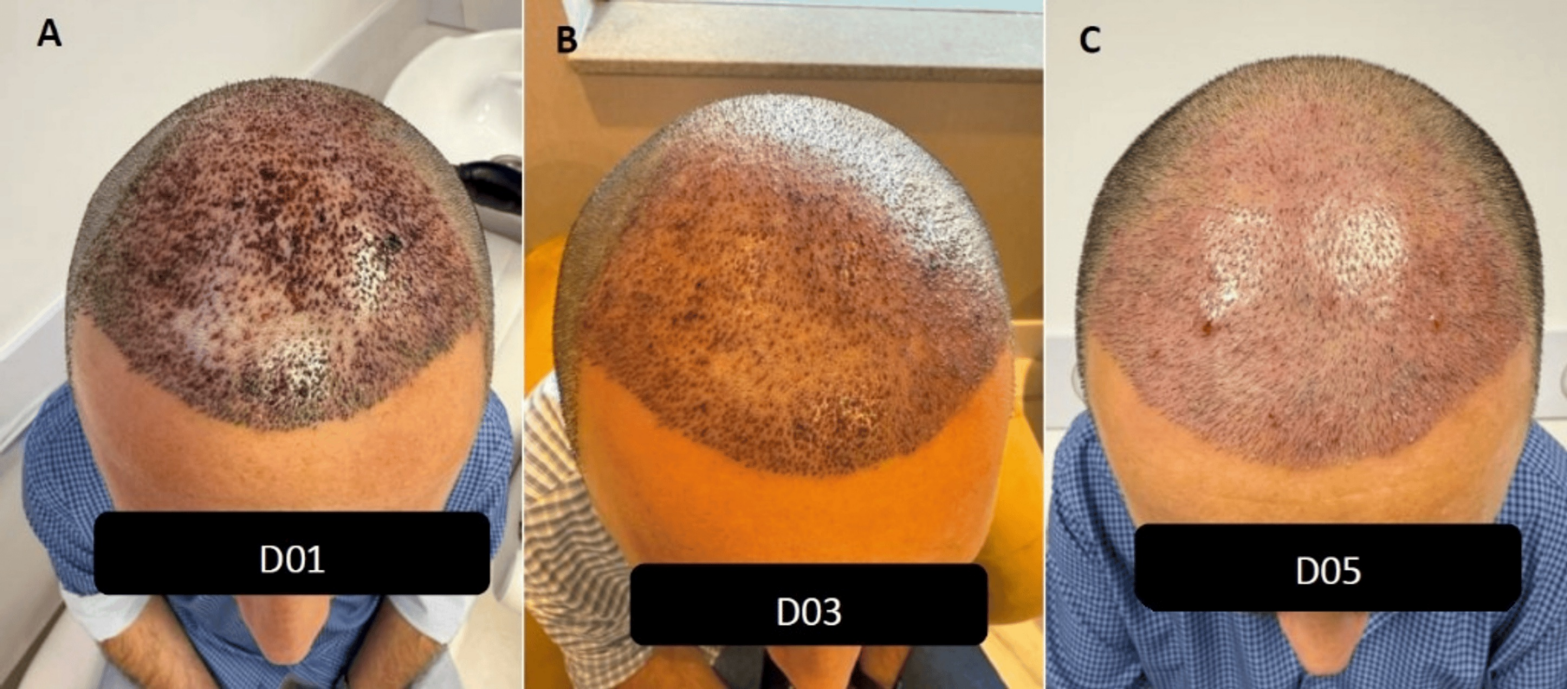
Case C (A) Day 1, pronounced crust formation and erythema. (B) Day 3, substantial reduction in crusting and inflammatory signs. (C) Day 5, complete crust resolution and uniform graft integration.

**Figure 4:**
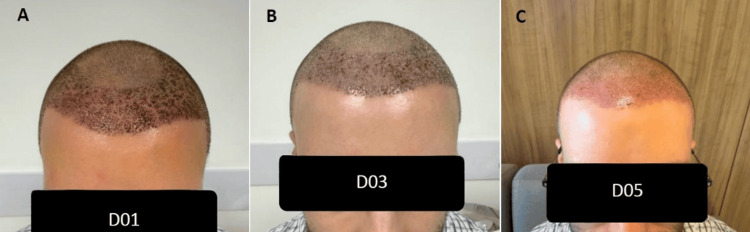
Case D (A) Day 1, moderate-to-severe crusting. (B) Day 3, partial crust resolution and reduced erythema. (C) Day 5, showing advanced healing with minimal residual crusting.

**Figure 5:**
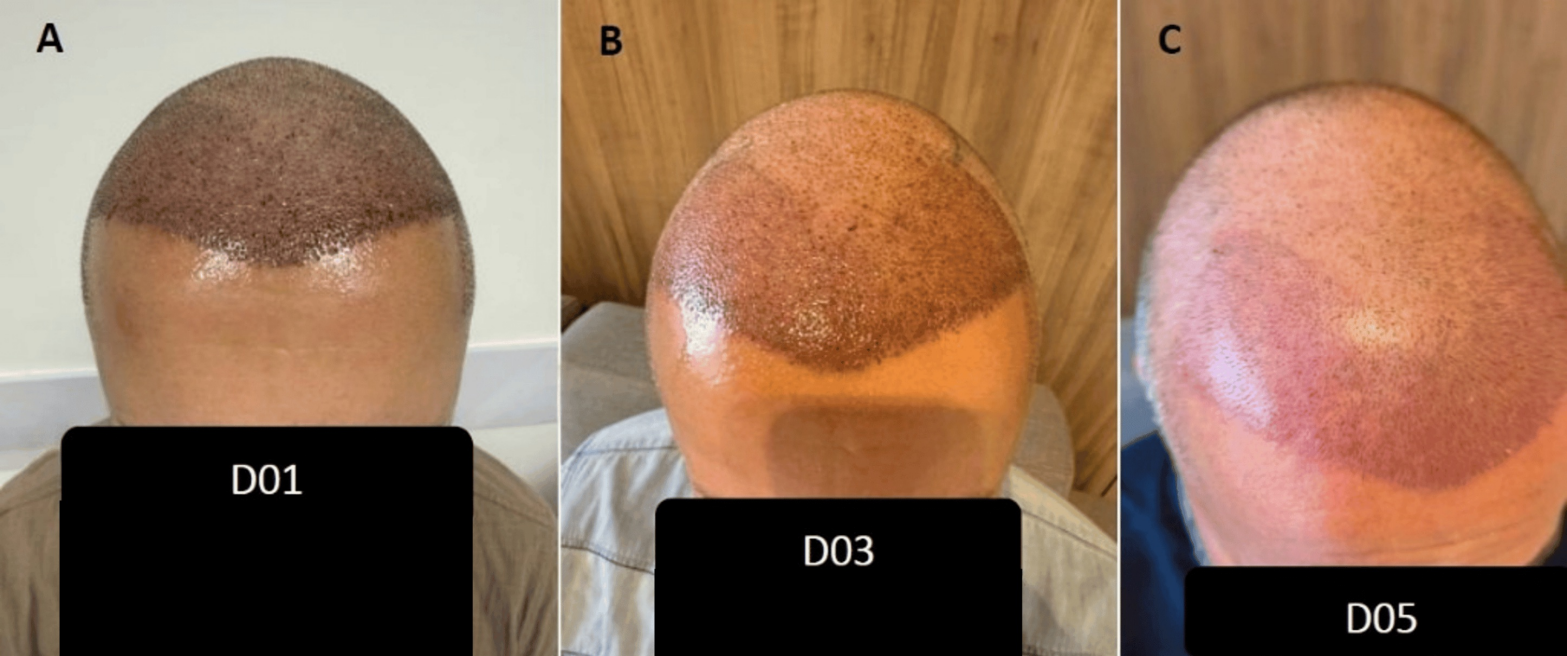
Case E (A) Day 1, showing moderate crust formation. (B) Day 3, with significant reduction in crusting. (C) Day 5, demonstrating complete crust resolution and favorable graft integration.

The most quantifiable outcome was the expedited resolution of postoperative crusting, which was documented through sequential photography (see Figures 1-5). Complete elimination of scab formation was achieved within a 5-day timeframe, which represents a marked improvement over typical recovery timelines in standard postoperative care protocols (Table 2). This accelerated crust resolution allowed for earlier visualization of the transplanted area and a more effective assessment of graft integration. The observed reduction in healing time facilitated the earlier return to normal hygiene routines for patients, potentially improving comfort and satisfaction during the recovery period. Additionally, the accelerated resolution of visible crusting may have psychosocial benefits, allowing patients to resume social and professional activities with minimal visible evidence of the recent procedure (Table 3). Table 4 approaches the pain assessment through a 10-point analog scale [11].

**Table 2:** Outcome assessment parameters

Patient	Resolution of crusting	Patient satisfaction	Recovery time	Complications
A	4 days	5	2	0
B	3 days	5	2	0
C	5 days	5	4	0
D	4 days	4	5	0
E	4 days	5	1	0

**Table 3:** Crusting/scabbing assessment (0-4 scale)

Patient	Day 1	Day 3	Day 5	Day 7
A	3	2	0	0
B	3	0	0	0
C	4	3	0	0
D	4	2	0	0
E	3	2	0	0

**Table 4:** Pain assessment (10-point analog scale)

Patient	Day 1	Day 2	Day 3	Day 4	Day 5	Day 6	Day 7
A	2	1	0	0	0	0	0
B	1	0	0	0	0	0	0
C	4	2	1	0	0	0	0
D	4	3	3	2	2	2	1
E	4	3	2	1	0	0	0

The photographic documentation demonstrates the progressive reduction in crusting/scabbing across all patients, with complete resolution by day five. Of particular note is the consistent pattern of healing observed across different patients, suggesting a reliable and reproducible effect of the HBOT protocol.

## Discussion

Hyperbaric chamber therapies have made significant contributions in various fields of medicine. Originally used for the treatment of decompression sickness, today we have an enormous field of applications focusing on wounds that are difficult to heal, necrotic and infectious lesions, osteomyelitis, and accidents such as burns and frostbite [2].

In the field of aesthetic surgery, we observe several benefits in the postoperative period such as accelerated healing, improved tissue perfusion, prevention of reperfusion injuries, prevention of tissue necrosis, increased rate of successful outcomes, and reduction of postoperative symptoms such as pain, pruritus, and edema, these related to the anti-inflammatory potential provided by hyperbaric oxygen [5-8,13,14].

Beyond accelerating healing and enabling complete resolution of the scab stage through earlier washing, postoperative HBOT has shown promising advances in the field of hair transplantation. One of the most promising benefits is the discovery that hyperbaric oxygen can reduce the shedding rate of transplanted hair follicles, as well as decrease the incidence of pruritus and folliculitis [1].

This discovery is related to the fact that HBOT directly increases the partial pressure of oxygen and enhances resistance to ischemia by improving tissue oxygenation, providing oxygen for normal metabolic activities. Furthermore, tissue ischemia found in the scalp during postoperative hair transplantation can increase the state of malnutrition that hair follicles experience due to their dependence on surrounding circulation. It is known that the use of HBOT promotes angiogenesis, and this earlier formation of blood vessels can lead to better oxygen and nutrient supply, reducing damage to transplanted hair follicles [1,6,7,15].

These findings suggest that HBOT may serve as a valuable adjunctive therapy in the immediate postoperative period following hair transplantation. The therapy's known mechanisms of enhanced tissue oxygenation, promotion of angiogenesis, and modulation of inflammatory responses may contribute to the observed acceleration in wound healing and crust resolution [2,5-7]. Controlled studies are warranted to establish optimal protocols and quantify the magnitude of benefit compared to standard care approaches [15].

Beyond the findings, the psychosocial significance of early scab removal in hair transplant postoperative care cannot be underestimated. The accelerated resolution of scabs through HBOT associated with earlier postoperative care protocols may significantly reduce the psychological burden that patients experience during the recovery phase. Furthermore, complete crust removal allows patients to resume social and professional activities with minimal visible evidence of the procedure. This fact deserves additional consideration: the reduction of "downtime" represents a significant improvement in quality of life that can influence the patient's decision-making about whether to undergo surgery amidst routine life and satisfaction with the final experience after the procedure. The potential of HBOT to shorten this socially limiting recovery period for some patients may represent an additional dimension of added value beyond the physiological benefits previously documented.

The cost involved in six sessions of hyperbaric oxygen therapy is equivalent to 5% of the total operational cost, considering surgery in a hospital center with all necessary equipment, which, in theory, is low given the benefits found and patient satisfaction rates.

Methodological limitations of the study are the small number of included patients and the absence of a control group, limiting the ability to perform robust statistical analyses.

## Conclusions

These preliminary findings from this case report suggest that hyperbaric oxygen therapy (HBOT) may be useful as a valuable adjunctive treatment in the postoperative management of hair transplantation patients. The accelerated wound healing observed in this study, with early resolution of crusting, proposes that HBOT has the potential to significantly reduce the recovery timeline compared with conventional postoperative protocols. These effects appear to work synergistically to support graft survival while simultaneously promoting faster recovery of the surrounding tissues. This faster healing process allowed patients to resume scalp hygiene practices earlier and facilitated a quicker return to daily activities.

Despite these encouraging outcomes, appropriate caution is warranted due to the inherent limitations of a case report. Larger, randomized controlled trials are necessary to confirm the efficacy of HBOT, determine the most effective timing and dosing protocols, and evaluate cost-effectiveness in the context of hair transplantation. Further studies should also explore whether benefits vary according to patient characteristics, transplantation techniques, or graft quantities. Nonetheless, these preliminary findings provide a strong rationale for the continued investigation of HBOT as a potentially valuable addition to postoperative care in hair restoration surgery.
